# Supplementary material for: Cerebrovascular risk factors impact frontoparietal network integrity and executive function in healthy ageing
Source: Nat Commun. 2020 Sep 7;11:4340. doi: 10.1038/s41467-020-18201-5 (PMC7477206; doi:10.1038/s41467-020-18201-5)
Supplement: Supplementary file 1 — Supplementary Information [file 41467_2020_18201_MOESM1_ESM.pdf]

Supplementary Information

Supplementary Table 1 | Population demographics

**Total N**	22059
**Sex**	
**Number Female (%)**	11128 (52)
**Number Male (%)**	10247 (48)
**Mean age (SD)**	61.7 (7.0)
**Number Midlife (44-69) (%)**	17399 (90)
**Number Late life (70+) (%)**	1956 (10)
**Median Townsend Index (IQR)**	-2.7 (3.2)
**Median years education (IQR)**	18 (6)
**Systolic blood pressure mean (SD) mmHg**	128 (25.5)
**Diastolic blood pressure mean (SD) mmHg**	88 (23)
**Blood pressure lowering medication**	
**ON (%)**	4699 (22)
**OFF (%)**	16410 (78)
**Blood pressure lowering medication (by age group)**	
**Mid-life ON (%)**	2656 (19)
**Mid-life OFF (%)**	11316 (81)
**Late-life ON (%)**	807 (34)
**Late-life ON (%)**	1543 (66)
**Cholesterol lowering medication**	
**ON (%)**	4509 (21)
**OFF (%)**	16600 (79)
**Smoking status**	
**Number never smoked (%)**	13966 (78)
**Number 10-50 pack years (%)**	2938 (17)
**Number >50 pack years (%)**	293 (1)
**Diagnosed diabetes**	
**YES (%)**	1098 (5)
**NO (%)**	20066 (95)
***APOE* ε Status**	
**Number ε3/3 (%)**	16854 (76)
**Number ε3/4 (%)**	4239 (19)
**Number ε4/4 (%)**	402 (2)
**Mean waist to hip ratio (SD)**	0.87 (0.1)
**Male**	0.93(0.06)
**Female**	0.81(0.07)
**Mean white matter hyperintensity load (SD) mm^3**	4467 (5621)

SD: standard deviation; IQR: interquartile range; APOE: apolipoprotein E.

Supplementary Table 2 | Biobank showcase variables used in all analyses

Variable	Field ID	Biobank showcase link	Instance
**Reaction time**	20023	http://biobank.ndph.ox.ac.uk/showcase/field.cgi?id=20023	2
**Pairs matching accuracy**	399	http://biobank.ndph.ox.ac.uk/showcase/field.cgi?id=399	2
**Pack years calculation**	20161	http://biobank.ctsu.ox.ac.uk/crystal/field.cgi?id=20161	2
**Ever smoked**	20160	http://biobank.ctsu.ox.ac.uk/crystal/field.cgi?id=20160	2
**Smoking status**	20116	http://biobank.ctsu.ox.ac.uk/crystal/field.cgi?id=20116	2
**Number of cigarettes smoked daily (current smokers)**	2887	http://biobank.ctsu.ox.ac.uk/crystal/field.cgi?id=2887	2
**Number of cigarettes smoked daily (previous smokers)**	6183	http://biobank.ctsu.ox.ac.uk/crystal/field.cgi?id=6183	2
**Age started smoking (current smokers)**		http://biobank.ctsu.ox.ac.uk/crystal/field.cgi?id=3436	2
	3436		
**Age started smoking (former smokers)**	2867	http://biobank.ctsu.ox.ac.uk/crystal/field.cgi?id=2867	2
**Ever tried to stop smoking**	3486	http://biobank.ctsu.ox.ac.uk/crystal/field.cgi?id=3486	2
**Stopped smoking (>6 months)**	2907	http://biobank.ctsu.ox.ac.uk/crystal/field.cgi?id=2907	2
**Age stopped smoking**	2897	http://biobank.ctsu.ox.ac.uk/crystal/field.cgi?id=2897	2
**Cholesterol lowering medication**	6177	http://biobank.ctsu.ox.ac.uk/showcase/field.cgi?id=6177	2
**Blood pressure medication**	6177	http://biobank.ctsu.ox.ac.uk/showcase/field.cgi?id=6177	2
**Diabetes- diagnosed by a doctor**	2443	http://biobank.ctsu.ox.ac.uk/showcase/field.cgi?id=2443	2
**Systolic blood pressure – automatic reading**	4080	http://biobank.ctsu.ox.ac.uk/showcase/field.cgi?id=4080	2
**Systolic blood pressure – manual reading**	93	http://biobank.ctsu.ox.ac.uk/showcase/field.cgi?id=93	2

**Diastolic blood pressure – automatic reading**	4079	http://biobank.ctsu.ox.ac.uk/showcase/field.cgi?id=4079	2
**Diastolic blood pressure – manual reading**	94	http://biobank.ctsu.ox.ac.uk/showcase/field.cgi?id=94	2
**Waist circumference**	48	http://biobank.ctsu.ox.ac.uk/crystal/field.cgi?id=48	2
**Hip circumference**	49	http://biobank.ctsu.ox.ac.uk/crystal/field.cgi?id=49	2
**White matter hyperintensity volume**	25781	http://biobank.ndph.ox.ac.uk/showcase/field.cgi?id=25781	NA

Supplementary Table 3 | Coordinates for nodes in frontoparietal control network derived by Yeo et al. [26]

Yeo et al., (2011) coordinates			Overlapping imaging derived phenotype	Field ID in Biobank Showcase
**X**	**Y**	**Z**		
-40	50	7	Frontal Pole	Right: 25783<br>Left: 25782
-43	-50	46	Angular Gyrus	Right: 25823<br>Left: 25822
-57	-54	-9	Middle temporal gyrus (temporo-occipital)	Right: 25807<br>Left: 25806
-5	22	47	Superior Frontal Gyrus	Right: 25787<br>Left: 25786
-6	4	29	Cingulate Gyrus	Right: 25839<br>Left: 25838
-4	-76	45	Precuneus cortex	Right: 25843<br>Left: 25842

Coordinates given in Montreal Neurological Institute (MNI) template space.

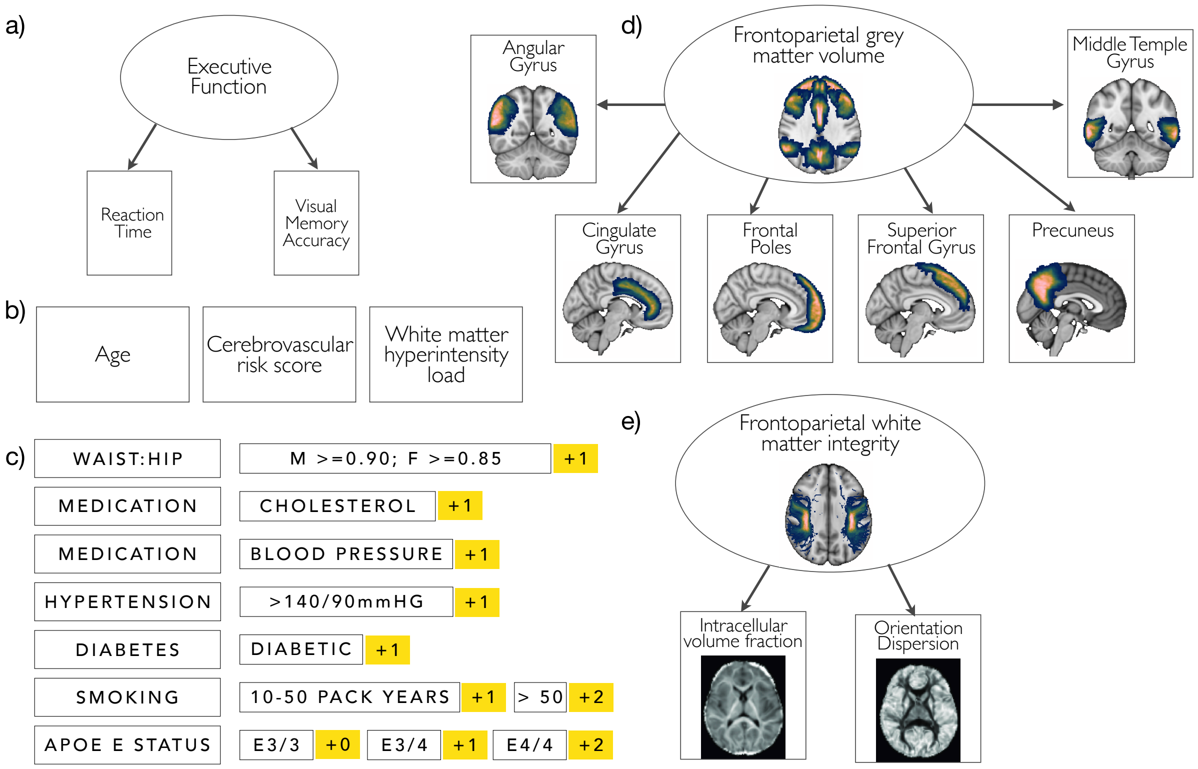

Supplementary Figure 1 | Cognitive, neuroimaging and cerebrovascular risk factor variables

a) Executive Function latent variable b) Observed variables in the structural equation model include age, white matter hyperintensity (WMH) load and cerebrovascular risk score which is a sum of the listed risk factors with the weightings shown in the yellow boxes (c). d) Regions of frontoparietal network included in the frontoparietal grey matter volume latent variable. e) Microstructural indices of the superior longitudinal fasciculus as indicators in the frontoparietal white matter integrity latent variable.

M: male; F: female; mmHg: millimetres of mercury; APOE: apolipoprotein E.

Supplementary Table 4 | Results of confirmatory factor analysis and path analysis. SLF- superior longitudinal fasciculus, CVR – cerebrovascular risk factor

Latent variable	Indicator	Standardised beta estimate	R^2	P-Value
Executive Function	Reaction time	0.49	0.24	0.0001
	Accuracy	0.25	0.06	0.0001
Frontoparietal white matter integrity	SLF Orientation Dispersion	0.25	0.06	0.0001
	SLF Intracellular Volume Fraction	0.65	0.42	0.0001
Frontoparietal grey matter volume	Frontal Pole	0.72	0.51	0.0001
	Angular Gyrus	0.38	0.15	0.0001
	Middle temporal gyrus (temporo-occipital)	0.34	0.12	0.0001
	Superior Frontal Gyrus	0.36	0.13	0.0001
	Cingulate Gyrus	0.13	0.02	0.0001
	Precuneus cortex	0.58	0.34	0.0001

Latent variable	Regressor	Standardised beta estimate	P-Value
Executive Function	Frontoparietal white matter integrity	0.32	0.0001
	Frontoparietal grey matter volume	0.34	0.0001
Frontoparietal white matter integrity	Age	-0.15	0.0001
	CVR score	-0.01	0.261
	White matter hyperintensity load	-0.52	0.0001
Frontoparietal grey matter volume	Age	-0.53	0.0001
	CVR score	-0.06	0.0001
	White matter hyperintensity load	-0.07	0.0001
White matter hyperintensity load	Age	0.36	0.0001
	CVR score	0.13	0.0001

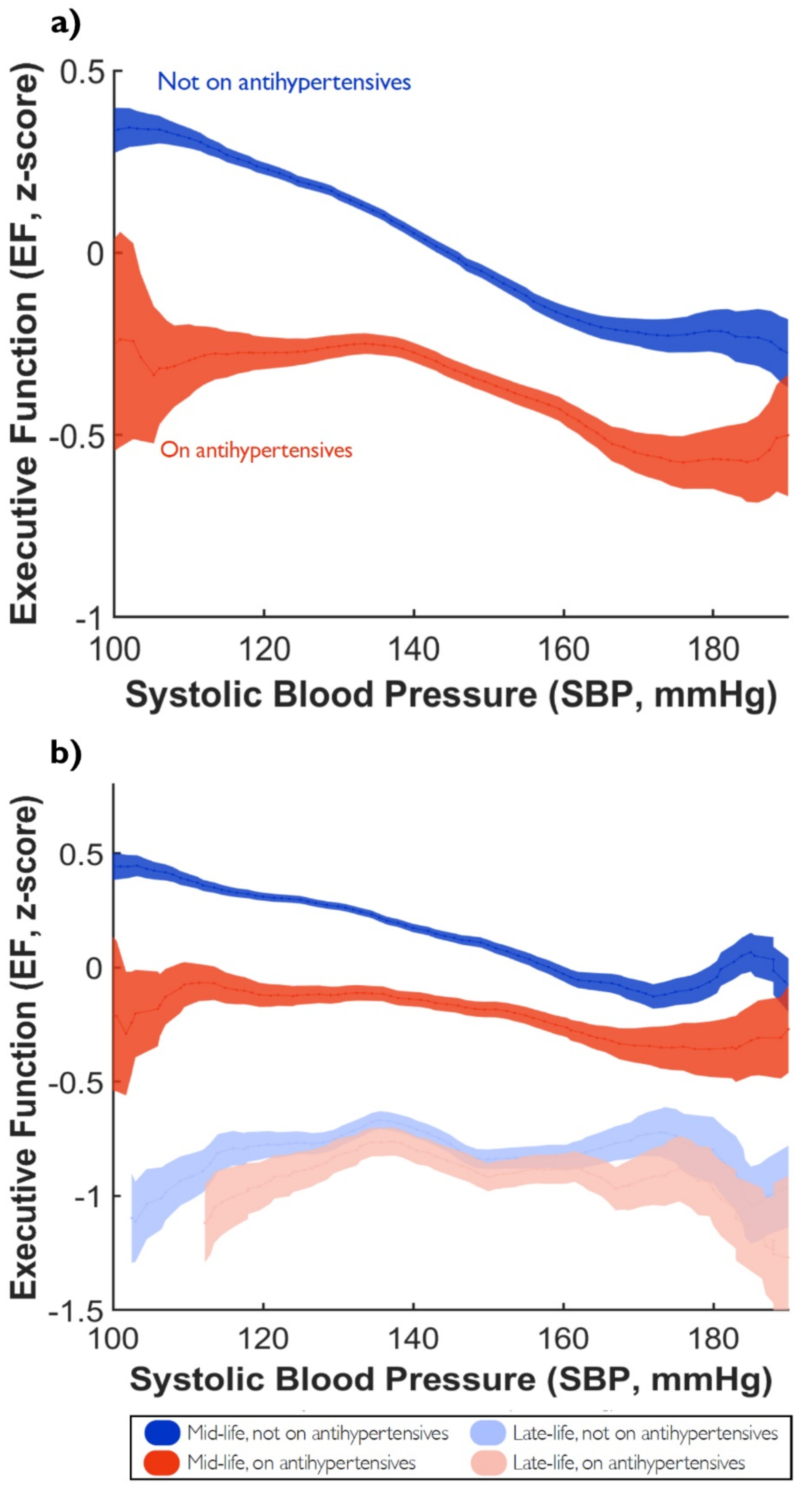

Supplementary Figure 2 | Sliding window curves with fixed systolic-bins against Executive Function latent variable. a) Compares participants on antihypertensives with those who are not. b) Examines participants on or not on antihypertensive in mid-life and late life groups. Shaded area represents standard error.

Supplementary note

Control network derivation and analysis

To test whether our structural model was specific to the frontoparietal control network we tested the same model but replaced the regional grey matter volumes and white matter tracts with those relevant to the language network. We used Neurosynth (Yarkoni *et al.*, 2011) to do a meta-analysis of 1101 studies using the term 'language' to identify regions of the brain consistently activated in language studies. From this meta-analysis we created a statistical map of the most consistently activated regions, thresholded at $p<0.01$ (false discovery rate). We visually inspected and manually selected the grey matter volume imaging derived phenotypes (IDPs) with the greatest overlap with the regions in the language meta-analysis mask. We chose the uncinate fasciculus and the inferior longitudinal fasciculus based on their established roles in the language network (Dick and Tremblay, 2012; Friederici and Gierhan, 2013; Dick *et al.*, 2014). Akaike Information Criterion (AIC) and Bayesian Information Criterion (Table 3) model fit indices, useful when two different non-nested models are compared, showed that indices for the control model (AIC= -642 894, BIC= -642589) were three times the magnitude of the full model (AIC=-214 446, BIC=-214 092).

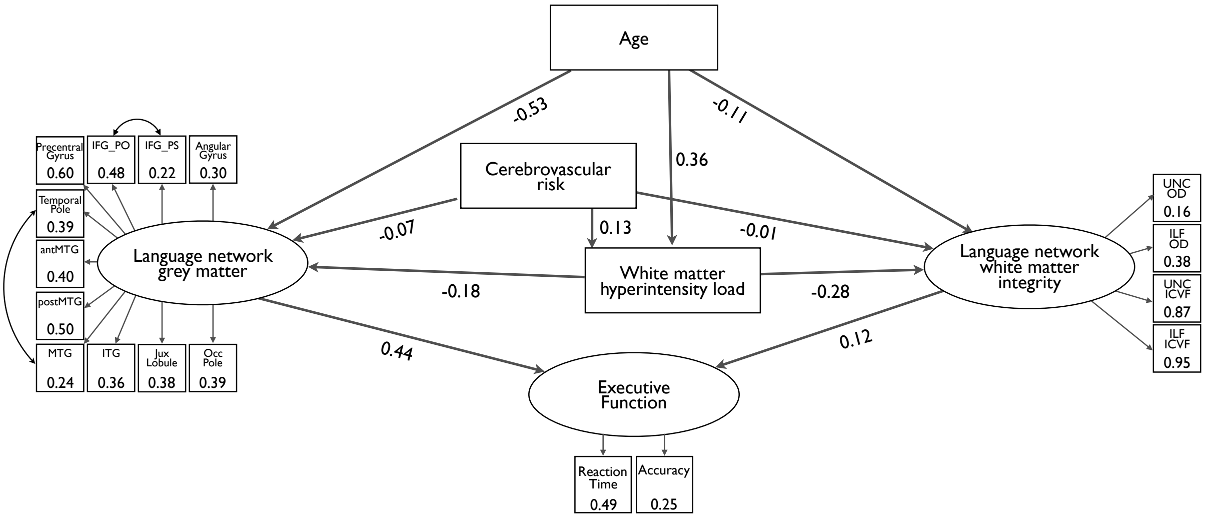

Supplementary Figure 3 | Control language network model. All values represent standardised beta estimates.

Abbreviations: ILF: inferior longitudinal fasciculus; UNC: uncinate fasciculus; IFG_PO: inferior frontal gyrus pars opercularis; IFG-PS: inferior frontal gyrus pars triangularis; antMTG: anterior middle temporal

gyrus; postMTG: posterior middle temporal gyrus; MTG: middle temporal gyrus; ITG: inferior temporal gyrus; Jux: Juxtapositional; Occ: occipital

Supplementary Table 5 | Model fit indices comparing nested and control networks

Model Fit Index		Interpretation	Model 1	Model 2	Model 3	Control Network
CFI	Comparative fit index	Higher is better; >0.9	0.92	0.74	0.81	0.82
TFI	Tucker-Lewis Index	Higher is better; >0.9	0.89	0.65	0.75	0.79
RMSE	Root mean square error of approximation	Lower is better; <0.06	0.04	0.08	0.06	0.06
AIC	Akaike Information Criterion	Lower is better	-214446	-209917	-211708	-642894
BIC	Bayesian Information Criterion	Lower is better	-214092	-209594	-211386	-642589

Model 1 is the full model. Model 2 is the nested model with constrained grey matter integrity. Model 3 is the nested model with white matter integrity constrained. The control network model replicated the full model using regions and tracts from a language network.
