# Supplementary material for: Cerebrovascular risk factors impact frontoparietal network integrity and executive function in healthy ageing
Source: Nat Commun. 2020 Sep 7;11:4340. doi: 10.1038/s41467-020-18201-5 (PMC7477206; doi:10.1038/s41467-020-18201-5)
Supplement: Supplementary file 3 — Reporting Summary [file 41467_2020_18201_MOESM3_ESM.pdf]

nature research

Corresponding author(s): Michele Veldsman

Last updated by author(s): 2020/07/27

Reporting Summary

Nature Research wishes to improve the reproducibility of the work that we publish. This form provides structure for consistency and transparency in reporting. For further information on Nature Research policies, see our Editorial Policies and the Editorial Policy Checklist.

Statistics

For all statistical analyses, confirm that the following items are present in the figure legend, table legend, main text, or Methods section.

n/a	Confirmed	
☐	☒	The exact sample size (n) for each experimental group/condition, given as a discrete number and unit of measurement
☒	☐	A statement on whether measurements were taken from distinct samples or whether the same sample was measured repeatedly
☐	☒	The statistical test(s) used AND whether they are one- or two-sided<br>*Only common tests should be described solely by name; describe more complex techniques in the Methods section.*
☐	☒	A description of all covariates tested
☐	☒	A description of any assumptions or corrections, such as tests of normality and adjustment for multiple comparisons
☐	☒	A full description of the statistical parameters including central tendency (e.g. means) or other basic estimates (e.g. regression coefficient) AND variation (e.g. standard deviation) or associated estimates of uncertainty (e.g. confidence intervals)
☐	☒	For null hypothesis testing, the test statistic (e.g. F, t, r) with confidence intervals, effect sizes, degrees of freedom and P value noted<br>*Give P values as exact values whenever suitable.*
☒	☐	For Bayesian analysis, information on the choice of priors and Markov chain Monte Carlo settings
☒	☐	For hierarchical and complex designs, identification of the appropriate level for tests and full reporting of outcomes
☐	☒	Estimates of effect sizes (e.g. Cohen's d, Pearson's r), indicating how they were calculated

Our web collection on statistics for biologists contains articles on many of the points above.

Software and code

Policy information about availability of computer code

Data collection	N/A
Data analysis	RStudio Version 1.1.456<br>MATLAB R2018a, conditional plotting:

For manuscripts utilizing custom algorithms or software that are central to the research but not yet described in published literature, software must be made available to editors and reviewers. We strongly encourage code deposition in a community repository (e.g. GitHub). See the Nature Research guidelines for submitting code & software for further information.

Data

Policy information about availability of data

All manuscripts must include a data availability statement. This statement should provide the following information, where applicable:

- Accession codes, unique identifiers, or web links for publicly available datasets
- A list of figures that have associated raw data
- A description of any restrictions on data availability

The data analysed during the current study are available from the UK Biobank https://www.ukbiobank.ac.uk/researchers/. The variables used are detailed in Table S2. Code for the sliding window analyses are available from https://osf.io/vmabg/.

Field-specific reporting

Please select the one below that is the best fit for your research. If you are not sure, read the appropriate sections before making your selection.

- [x] Life sciences
- [ ] Behavioural & social sciences
- [ ] Ecological, evolutionary & environmental sciences

For a reference copy of the document with all sections, see nature.com/documents/nr-reporting-summary-flat.pdf

Life sciences study design

All studies must disclose on these points even when the disclosure is negative.

Sample size	Data from 22059 individuals were included in the study. These were the maximum number of people with imaging data available in the UK Biobank at the time of analysis. After exclusions (see below) 20 460 individuals were included in the analysis.
Data exclusions	We excluded individuals with a history or current diagnoses of neurological disease, brain injury, stroke, transient ischaemic attack, subdural or subarachnoid haematoma; infection of the nervous system; brain abscess, haemorrhage or skull fracture; encephalitis, meningitis, chronic neurological problem, amyotrophic lateral sclerosis, multiple sclerosis, Parkinson's or Alzheimer's disease, epilepsy, head injury, alcohol, opioid and other dependency according to the non-cancer illnesses codes (http://biobank.ndph.ox.ac.uk/showcase/coding.cgi?id=6).
Replication	The main model was tested against a control model (supplementary information) to ensure specificity of the model and that it could not be reproduced with an irrelevant brain network.
Randomization	The majority of our analysis were conducted across the entire population. No randomization was necessary as we were looking at continuous variables across the population.
Blinding	Blinding was not relevant to this study as analyses included the entire population

Reporting for specific materials, systems and methods

We require information from authors about some types of materials, experimental systems and methods used in many studies. Here, indicate whether each material, system or method listed is relevant to your study. If you are not sure if a list item applies to your research, read the appropriate section before selecting a response.

Materials & experimental systems

n/a	Involved in the study
x	Antibodies
x	Eukaryotic cell lines
x	Palaeontology and archaeology
x	Animals and other organisms
	x Human research participants
x	Clinical data
x	Dual use research of concern

Methods

n/a	Involved in the study
x	ChIP-seq
x	Flow cytometry
	x MRI-based neuroimaging

Human research participants

Policy information about studies involving human research participants

Population characteristics	Clinical, demographic, cognitive and brain imaging data were analysed in 22,059 people from the UK Biobank aged 44-73 (mean age 62, SD 7) at the time of their MRI scan. 52% of the population were female.
Recruitment	Participants were recruited by UK Biobank. 9.2 million individuals were invited to take part aged 40-69 years and living within 40km of the assessment centres across the UK. Detailed investigations of the representativeness of the sample has revealed a bias towards healthier, more educated and higher socio-economic status than the general population.
Ethics oversight	Research Ethics Committee (REC number 11/NW/0382)

Note that full information on the approval of the study protocol must also be provided in the manuscript.

Magnetic resonance imaging

Experimental design

Design type	Multimodal imaging

Design specifications | Imaging derived phenotypes (IDPs) were used in the analyses. All preprocessing and analyses were conducted according to the UK Biobank imaging pipeline. Details of the generation of IDPs is available here: https://doi.org/10.1016/j.neuroimage.2017.10.034

Behavioral performance measures | N/A

Acquisition

Imaging type(s) | structural, diffusion

Field strength | 3T

Sequence & imaging parameters | Sequence and imaging parameters are openly available here: https://biobank.ctsu.ox.ac.uk/crystal/crystal/docs/brain_mri.pdf

Area of acquisition | Whole brain

Diffusion MRI ☒ Used ☐ Not used

Parameters | Mu,ti-band, 100 encoding directions (50 b=1000 s mm, 50 b-2000 s mm)

Preprocessing

Preprocessing software | The full processing pipeline is openly available here: https://doi.org/10.1016/j.neuroimage.2017.10.034

Normalization | see above

Normalization template | MNI 152

Noise and artifact removal | see above

Volume censoring | see above

Statistical modeling & inference

Model type and settings | n/a

Effect(s) tested | n/a

Specify type of analysis: ☐ Whole brain ☒ ROI-based ☐ Both

Anatomical location(s) | Structural: FSL FAST segmentation
Diffusion: John Hopkins University tractography atlas

Statistic type for inference (See Eklund et al. 2016) | n/a

Correction | see above

Models & analysis

n/a	Involved in the study
☒	☐ Functional and/or effective connectivity
☒	☐ Graph analysis
☒	☐ Multivariate modeling or predictive analysis
